# Ginsenosides Rg1 from *Panax ginseng*: A Potential Therapy for Acute Liver Failure Patients?

**DOI:** 10.1155/2014/538059

**Published:** 2014-10-28

**Authors:** Jinqiu Zhao, Zhengyu Shi, Shu Liu, Jiajun Li, Wenxiang Huang

**Affiliations:** Department of Infectious Diseases, The First Affiliated Hospital of Chongqing Medical University, Yuzhong District, Chongqing 400016, China

## Abstract

Acute liver failure (ALF) is a rapidly progressing critical illness with a high mortality rate. Circulating inflammatory cytokines, such as tumor necrosis factor-*α* (TNF-*α*), play a significant role in the pathophysiology of ALF through promoting hepatocellular apoptosis. Ginsenoside Rg1, the primary active ingredient in* Panax ginseng* (also termed Asian or Korean ginseng), has been reported to inhibit TNF-*α* production and has been shown to significantly attenuate liver fibrosis development. Here, we assessed ginsenoside Rg1's potential as a therapy for ALF by investigating the effect of ginsenoside Rg1 treatment on circulating inflammatory markers, hepatocellular apoptosis, and relevant apoptotic signaling pathways in a well-established murine ALF model. We found that ginsenoside Rg1 significantly reduces liver damage in a murine ALF model through inhibiting TNF-*α*-induced, caspase-dependent hepatocellular apoptosis. These results support the further investigation of ginsenoside Rg1 as a therapeutic candidate for ALF.

## 1. Introduction

Acute liver failure (ALF) is a rapidly progressing critical illness with a high mortality rate [1]. Although liver transplantation is the best current treatment for ALF, this option is limited by insufficient donors and high treatment costs [2]. Therefore, new therapeutic strategies are urgently needed for ALF. However, achieving this goal has been hindered by our poor understanding of the pathophysiological mechanisms underlying ALF [3].

There is increasing evidence that activation of systemic immune responses plays a pivotal role in ALF pathogenesis and outcomes [4]. In particular, circulating inflammatory cytokines, such as tumor necrosis factor *α* (TNF-*α*), interleukin (IL)-1*β*, and IL-6, play a significant role in the pathophysiology of ALF. For example, TNF-*α* in the acute phase response is significantly elevated in the livers and sera of ALF patients, and IL-6 has been shown to ameliorate acute liver injury in murine models of ALF through downregulating TNF-*α* production [4]. On a hepatocellular level, circulating inflammatory cytokines such as TNF-*α* appear to promote apoptosis [5, 6]. After TNF-*α* binding, an intracellular region of the hepatocellular TNF-R1 receptor (termed the “death domain”) recruits TNF receptor-associated protein with death domain (TRADD). TNF-R1-bound TRADD then serves as an assembly platform for binding of TNF-*α* receptor-associated factor (TRAF)2, receptor-interacting kinase (RIP), and the adapter molecule Fas-associated death domain (FADD). TNF-*α*-induced TNF-R1 activation through RIP1 and TRAF2 can lead to downstream nuclear factor-kappaB (NF-*κ*B) activation (which plays a role in regulating apoptosis), whereas TNF-*α*-TNF-R1 activation through FADD can activate downstream caspases that lead to apoptosis.

Ginsenoside Rg1 (G-Rg1) is the primary pharmacologically active compound in* Panax ginseng* (also termed Asian or Korean ginseng) [7]. A wide range of trophic and protective effects have been reported for G-Rg1, including angiogenesis [8], neuroprotection [9], and progenitor cell proliferation [10, 11]. With respect to this study, G-Rg1 has been shown to inhibit the production of TNF-*α* [12] and has been shown to significantly attenuate the development of liver fibrosis [13]. Therefore, G-Rg1 treatment may produce antiapoptotic or proliferative effects in hepatocytes that can be effective in treating ALF.

In this study, we aimed to assess G-Rg-1's potential as a therapy for ALF by analyzing its use in a well-established CCl_4_-induced ALF model. Specifically, we investigated whether G-Rg1 treatment has an effect on circulating inflammatory markers. We also assessed the effects of G-Rg1 treatment on hepatocellular apoptosis and relevant apoptotic signaling pathways.

## 2. Materials and Methods

### 2.1. Ethics Statement

All the following procedures were strictly consistent with 3R principles, while animal care and housing procedures were in compliance with Chinese regulatory requirements. The protocol of this study was approved prior to implementation by the Ethics Committee of Chongqing Medical University, and all procedures were in accordance with the National Institutes of Health Guidelines for Animal Research (Guide for the Care and Use of Laboratory Animals). Special care was taken to minimize number of and suffering of animals.

### 2.2. Animals and Model Construction

A total of 160 male C57 BL/6 mice (weights: 20–22 g; age: eight weeks) were purchased from the Animal Facility at Chongqing Medical University (Chongqing, China). The mice were randomly divided into four groups (*n* = 40 per group): an ALF group, a G-Rg1_pre-ALF_ group, a G-Rg1_post-ALF_ group, and a control (CON) group. The ALF model was constructed by hypodermic injection of 0.2 mL CCl_4_ in olive oil (olive oil: CCl_4_ = 3 : 1) into the ALF, a G-Rg1_pre-ALF_, and G-Rg1_post-ALF_ groups. The ALF group was left untreated. The G-Rg1_pre-ALF_ group was given 0.2 mL G-Rg1 (diluted with distilled water to 4 mg/mL) by intragastric administration twice a day prior to ALF induction, while the G-Rg1_post-ALF_ group was given 0.2 mL G-Rg1 (diluted with distilled water to 4 mg/mL) after ALF induction. The CON group was administered the same volume of saline in the identical manner.

Serum was collected from all mice 24 h prior to sacrifice for later biochemical testing and enzyme-linked immunosorbent assay (ELISA). After sacrifice, the liver was immediately excised and part of it was stored at −80°C for later Western blotting, whereas another part was fixed in 10% formalin for later histopathology and immunohistochemistry.

### 2.3. ELISA

ELISA kits specific for mouse IL-6, IL-1*β*, and TNF-*α* (USCN) were used according to the manufacturer's instructions. ELISA was performed in triplicate for each sample. Serum levels of TB, ALT, and AST (Nanjing Jiancheng Biological Technology, China) were measured according to the manufacturer's instructions.

### 2.4. Histology and Immunohistochemistry

Liver samples were fixed for 24 h in 4% paraformaldehyde at 4°C and then processed by paraffin embedding according to standard methods. Consecutive 4 *μ*m sections were performed using a rotary microtome (Leica RM 2135, Meyer Instruments, Houston, TX, USA) for hematoxylin and eosin (HE) staining, terminal deoxynucleotidyl transferase dUTP nick end labeling (TUNEL) staining, and histochemistry.

Immunohistochemical staining was performed using the SP immunohistochemistry kit according to the manufacturer's instructions. Briefly, sections were dewaxed with xylene and rehydration, and then microwave antigen retrieval was performed at 95°C for 15 min by using 0.01 M citric acid buffer (pH 6.0). The sections were then incubated with a 3% hydrogen peroxide solution for 10 min at room temperature (RT). Following several washes in PBS (pH 7.4), sections were blocked with 10% goat serum and then incubated overnight with polyclonal rabbit anti-rat Ab1-42 antibody (1 : 600), PPARc antibody (1 : 400), and IDE antibody (1 : 1300) at 4°C. After several washes with PBS, biotinylated anti-mouse IgG or anti-rabbit IgG (secondary antibody) was added at 37°C for 30 min. Horseradish peroxidase-labeled streptavidin was added after several washes with PBS, followed by 30 min of incubation at 37°C. After additional washes with PBS, immunoreactivity was detected with a diaminobenzidine (DAB) staining kit, and sections were counterstained with HE.

### 2.5. *In Situ* TUNEL Staining

The TUNEL assay was performed using the ApopTag1 Plus Peroxidase* In Situ* Apoptosis Kit (catalog #S7101; Millipore, Danvers, Massachusetts, USA) according to the manufacturer's instructions with minor modifications. Nonspecific labeling in the sections was prevented with 3% bovine serum albumin (BSA) and 20% normal bovine serum in PBS for 30 min at room temperature. Terminal deoxynucleotidyl transferase (TdT) and fluorescein-dUTP (TUNEL reaction mixture) were added to the sections before incubating the sections in a dark humidified chamber at 37°C for 60 min. After PBS rinsing, sections were mounted and counterstained with the fluorescent dye 4′-6-diamidino-2-phenylindole (DAPI) for visualization of the nuclei. Three randomly selected regions from each cross-section were digitally recorded with a CCD camera (Olympus, Melville, NY) to visualize TUNEL-positive nuclei using an Olympus fluorescence microscope (Melville, NY) with a 20x magnification. CON group experiments were performed in parallel using DNase 1 (with and without TdT) to verify the specificity of labeling.

### 2.6. Western Blotting

Livers (*n* = 2 per group) were homogenized in a standard lysis buffer and then sonicated to promote lysis. Western blotting was performed in triplicate for each sample. The samples were centrifuged at 12000 rpm at 4°C for 10 min. The supernatants were collected, and protein amounts were quantified using the BAC method. Lysates containing equal amounts of protein were boiled at 95°C in SDS sample buffer for 5 min, electrophoresed on 10% denaturing sodium dodecylsulphate-polyacrylamide gel electrophoresis (SDS-PAGE), transferred to PVDF membranes (Millipore), blocked with 5% skimmed milk for 1 h at room temperature, and incubated overnight at 4°C with the following primary polyclonal antibodies: rabbit anticleaved caspase-3 (Asp175) (5A1E) (1 : 1000, CST 9664S, Proteintech), rabbit anticaspase-8 (1 : 200, Abcam), and rabbit anti-NF-*κ*B p65 (1 : 200, Abcam). After three 30 min rinses in 100 mM Tris-HCl buffer (pH 7.5) with 150 mM NaCl and 0.05% Tween 20 (TTBS buffer), the membranes were incubated at 37°C for 60 min with a HRP-conjugated secondary antibody (1 : 3000, Jackson). Finally, the proteins were detected with electrochemiluminescence (ECL) and exposed to X-ray film (Kodak, USA). Immunoreactive bands were quantified with Bio-Rad Quantity One software (Bio-Rad). Values were normalized based on the density values of the internal standard *β*-actin.

### 2.7. Electrophoretic Mobility Shift Assay (EMSA) for NF-*κ*B

EMSA was performed using the LightShift Chemiluminescent EMSA Kit (Pierce Biotechnology, Rockford, IL, USA) according to the manufacturer's instructions. All steps were performed on ice. Cells were harvested from 100 mm culture dishes using 1 mL PBS and transferred to microcentrifuge tubes (2500 rpm, 4 min, 4°C). Cells were washed with PBS and combined two 100 mm culture dishes per sample (2500 rpm, 4 min, 4°C). Cell pellets were resuspended with 1 mL lysis buffer, placed on ice for 5 min, and then centrifuged (2500 rpm, 4 min, 4°C). Nuclear pellets were washed with 1 mL lysis buffer (without NP-40), resuspended in 50–100 *μ*L of nuclear extract buffer, placed on ice for 10 min, vortexed briefly, and centrifuged (2500 rpm, 4 min, 4°C). Aliquots (10–20 *μ*L) of the supernatant containing nuclear extracts were quickly frozen on liquid nitrogen and then stored at −80°C.

Meanwhile, polyacrylamide minigels (8.0 × 8.0 × 0.1 cm) were optimized for detection. A 5% polyacrylamide gel was prepared, polymerized for at least 1 h, and then prerun for 2 h at 150 V. A gel loading sample was prepared and incubated the materials for 10 min at room temperature. Then, 1 *μ*L of competitive unlabeled NF-*κ*B p65 oligonucleotide probes (M-P65-3: 5′-TCGACAGAGGGACTTTCCGAGAGGC-3′; M-P65-4: 5′-TCGAGCCTCTCGGAAAGTCCCTCTG-3′) was added to the inducer group and incubated for 15 min at room temperature. Meanwhile, 2 *μ*L of biotin-labeled NF-*κ*B p65 oligonucleotide probes (M-P65-1: 5′-TCGACAGAGGGACTTTCCGAGAGGC-3′-biotin; M-P65-2: 5′-TCGAGCCTCTCGGAAAGTCCCTCTG-3′-biotin) was added and incubated for 25 min at room temperature. Then, 10 *μ*L of sample was loaded into the 5% polyacrylamide gel and run for 3 h at 150 V (gel running buffer: 0.25x TBE (pH 8.0)) and then transferred to a positive nylon membrane. The nylon membranes were UV cross-linked, probed with streptavidin-HRP conjugate, and incubated with the chemiluminescent substrate for chemiluminescent detection according to the manufacturer's instructions (LightShift Chemiluminescent EMSA Kit, Pierce Biotechnology, Rockford, IL, USA).

### 2.8. Statistical Analysis

All quantitative values were presented as means ± SDs. To rule out survival rate differences across the four groups, Chi-square testing was used. One-way ANOVA was performed in comparisons between the four groups. The post hoc Bonferroni multiple-comparison test was employed to compare among each paired group. *P* values of less than 0.05 were deemed to be statistically significant for all analyses. All data management and statistical analysis were performed using Stata 12.0 (StataCrop LP, College Station, Texas, USA).

## 3. Results

### 3.1. Animal Survival

After CCl_4_ injection, the 24-hour survival rate was 55% in the ALF group (Table 1). The two groups that received G-Rg1 treatment showed higher survival rates with a 75% survival rate in the G-Rg1_Pre-ALF_ group and a 80% survival rate in the G-Rg1_Post-ALF_ group. There was a significant difference in survival rates between the ALF group and the G-Rg1_Post-ALF_ group (*P* = 0.017). The survival rate of the CON group was 100%.

### 3.2. Serum Levels of TB, ALT, AST, IL-6, IL-1*β*, and TNF-*α*

To examine G-Rg1's hepatoprotective effects, we analyzed serum TB, ALT, AST, IL-6, IL-1*β*, and TNF-*α* as indicators of liver injury. There were significant differences in their plasma levels between the ALF and CON groups (Table 2). Compared with the ALF group, TB, ALT, and AST rapidly decreased after G-Rg1 treatment (*P* < 0.05). In addition, following CCl_4_ stimulation, G-Rg1 intervention significantly reduced the secretion of IL-6, IL-1*β*, and TNF-*α* compared to the ALF group.

### 3.3. Liver TUNEL Staining

**Figure 1:**
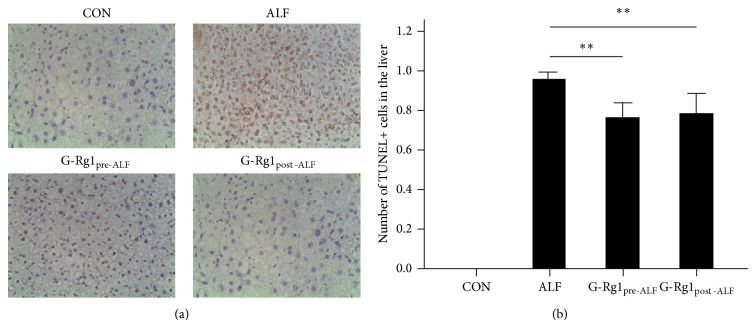
Terminal deoxynucleotidyl transferase dUTP nick end labeling (TUNEL) staining. (a) TUNEL+ cells were wildly distributed in the ALF group, but very few were observed in the CON group (all fields: 10x magnification). (b) After G-Rg1 treatment, the number of TUNEL-positive cells in both the G-Rg1_Pre-ALF_ and G-Rg1_Post-ALF_ groups was significantly less than that of the ALF group (^**^ *P* < 0.01). The data graphed in (b) are not directly representative of the field in (a); in order to make the graph more statistically robust, (b) displays the average number of TUNEL+ cells for each experimental group derived from sampling 10 independent fields of view for each experimental group under 40x magnification.

To detect the antiapoptotic effects of G-Rg1 treatment, liver tissues were analyzed for TUNEL-positive cells. TUNEL-positive cells were wildly distributed in the ALF group, but very few were observed in the CON group (Figure 1). After G-Rg1 treatment, the number of TUNEL-positive cells in both the G-Rg1_Pre-ALF_ and G-Rg1_Post-ALF_ groups was significantly less than that of the ALF group (*P* < 0.001) (Figure 1).

### 3.4. Caspase-3 and Caspase-8 Expression

**Figure 2:**
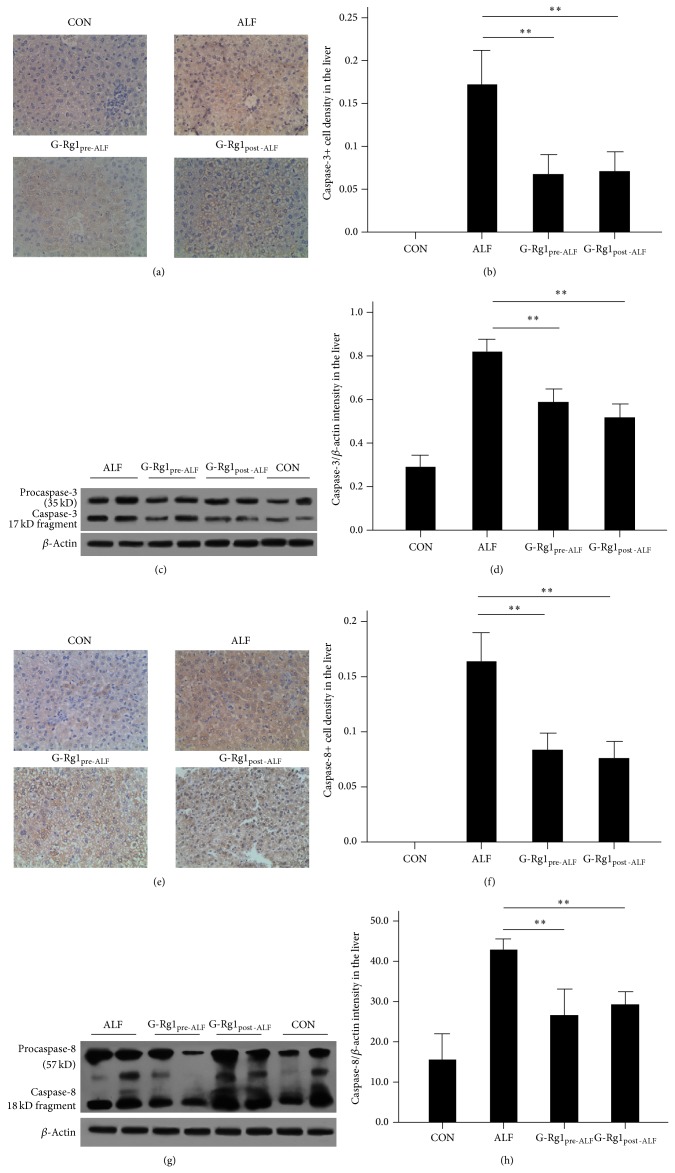
Caspase-3 and caspase-8 expression. Representative photographs of immunohistochemical staining of (a) caspase-3 and (e) caspase-8 as well as Western blots of (c) caspase-3 and (g) caspase-8 protein expression. Immunohistochemical staining showed that (b) caspase-3 levels and (f) caspase-8 expression levels were significantly increased in the ALF group as compared to the CON group (^**^ *P* < 0.01). The immunohistochemical staining data graphed in (b) and (f) are not directly representative of the fields in (a) and (e); in order to make the graphs more statistically robust, (b) and (f) display the average optical density (OD) values for each experimental group derived from sampling 10 independent fields-of-view for each experimental group. Western blotting also showed that that (d) caspase-3 and (h) caspase-8 expression levels were significantly increased in the ALF group as compared to the CON group (^**^ *P* < 0.01). The graph in (d) represents the OD of the caspase-3 17 kD fragment's band, and the graph in (h) represents the OD of the caspase-8 18 kD fragment's band. By both immunohistochemistry and Western blotting, this upregulation of caspase-3 and caspase-8 expression was partially reversed by G-Rg1 treatment (^**^ *P* < 0.01). Note: normal cells also display procaspase-3 and procaspase-8 expression as well as activated caspase-3 and caspase-8 expression, so there were small amounts of these bands in the control group.

To explore a possible caspase-based mechanism for this antiapoptotic effect, immunohistochemical and Western blot analyses on caspase-3 and caspase-8 expression were conducted. Levels of caspase-3 and caspase-8 were significantly increased in the livers of the ALF group as compared to the CON group (*n* = 5, *P* < 0.01 versus CON; Figure 2). This caspase upregulation was partially reversed by G-Rg1 treatment (*n* = 5, *P* < 0.01 versus ALF; Figure 2).

### 3.5. NF-*κ*B p65 DNA Binding Activity and Expression

**Figure 3:**
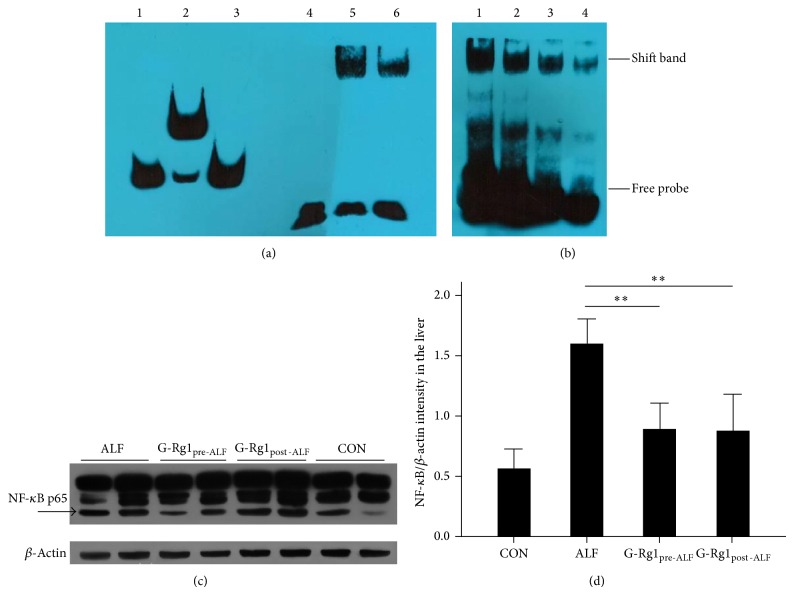
NF-*κ*B p65 DNA binding activity and expression. (a) Electrophoretic mobility shift assay (EMSA): bands 1–3 contain Epstein-Barr nuclear antigen- (EBNA-) based controls from the LightShift Chemiluminescent EMSA Kit (Thermo Scientific, USA); band 1, biotin-EBNA control DNA that establishes the position of an unshifted probe band; band 2, biotin-EBNA control DNA + EBNA extract containing sufficient target protein to shift the biotin-EBNA DNA; and band 3, biotin-EBNA control DNA + EBNA extract + 200-fold excess of unlabeled EBNA DNA demonstrating signal shift is prevented by competition from excess nonlabeled DNA. Band 4 contains only biotin-labeled probes. Band 5 contains biotin-labeled probes and nucleoprotein from normal liver cells. Band 6 contains biotin-labeled probes, unlabeled probes, and nucleoprotein from normal liver cells; the biotin-labeled and unlabeled probes compete in binding to the nucleoprotein. Band 6's signal is weaker than band 5's signal, since a lesser amount of competitively binding unlabeled probes was added to band 6. (b) EMSA: the ALF group (band 1) had the highest density shift band. The G-Rg1_pre-ALF_ group (band 2) showed a lower density shift band followed by the G-Rg1_post-ALF_ group (band 3) and the CON group (band 4). NF-*κ*B p65 DNA binding activity was the highest in the ALF group. G-Rg1 treatment significantly reduced NF-*κ*B p65 DNA binding activity in both the G-Rg1_Pre-ALF_ and G-Rg1_Post-ALF_ groups relative to the ALF group. (c, d) Western blotting revealed significant decreases in NF-*κ*B p65 expression in the G-Rg1_Pre-ALF_ and G-Rg1_Post-ALF_ groups relative to the ALF group (*n* = 5, ^**^ *P* < 0.01). The graph in (d) displays the average OD values for NF-*κ*B p65 for each experimental group derived from Western blotting experiments run in triplicate.

We studied whether G-Rg1 treatment differentially affected NF-*κ*B p65 DNA binding activity as measured by EMSA. There was a significant induction of NF-*κ*B p65 DNA binding activity in the ALF group relative to the CON group (Figure 3). G-Rg1 treatment significantly decreased NF-*κ*B p65 DNA binding activity in both the G-Rg1_Pre-ALF_ and G-Rg1_Post-ALF_ groups relative to the ALF group. By Western blot analysis, significant decreases in NF-*κ*B p65 expression were observed in the G-Rg1_Pre-ALF_ and G-Rg1_Post-ALF_ groups relative to the ALF group (*n* = 5, *P* < 0.01; Figures 3(c) and 3(d)).

## 4. Discussion

The CCl_4_-induced ALF model constructed here has been well-established in investigations of signal transduction and cell cycle events* in vivo* [14, 15]. Compared to the CON group, the ALF group showed significantly decreased survival accompanied by a significant elevation in serum ALT, AST, and TB levels; these proteins are released from damaged hepatocytes and are clinical indicators of acute hepatic injury [16]. G-Rg1 administration improved survival in both G-Rg1 groups but slightly favored the G-Rg1_Post-ALF_ group (80% versus 75%) (Table 1). G-Rg1 treatment significantly reduced serum ALT, AST, and TB elevation in both G-Rg1 groups, indicating that G-Rg1 treatment (both before and after ALF induction) has a protective role on hepatocytes (Table 2). In accordance, TUNEL images showed that G-Rg1 administration significantly inhibited hepatocellular death and destruction of liver architecture in both G-Rg1 groups (Figure 1).

To investigate the mechanism(s) underlying G-Rg1's beneficial effects, serum levels of IL-1*β*, IL-6, and TNF-*α* were analyzed, as these are acute-phase proteins that are considered to be systemic biomarkers of inflammation [17]. The ALF group showed significant elevations in serum IL-1*β*, IL-6, and TNF-*α* levels relative to the CON group (Table 2). Notably, G-Rg1 treatment significantly decreased serum levels of IL-1*β*, IL-6, and TNF-*α* expression in both G-Rg1 groups, indicating that G-Rg1 treatment (both before and after ALF induction) inhibits the production of these systemic inflammatory biomarkers (Table 2).

TNF-*α*-induced hepatocellular apoptosis is a key mechanism underlying liver injury in ALF [18], in which the caspase family plays a key role. TNF-*α*-induced activation of caspase-8, an initiator (or apical) caspase that is activated by a variety of apoptotic signals [19], has been previously implicated in hepatocellular apoptosis [20]. Activated caspase-8 cleaves and activates effector caspases, such as caspase-3, which, in turn, activates a variety of downstream targets. Here, the ALF group displayed significant upregulation of proapoptotic caspase-3 and caspase-8 expression relative to the CON group (Figure 2). Consistent with the TNF-*α* findings, G-Rg1 treatment significantly downregulated both caspase-3 and caspase-8 expression in both G-Rg1 groups (Figure 2), indicating that G-Rg1 treatment (both before and after ALF induction) inhibits the caspase-8/3 signaling cascade.

The TNF-*α*-induced, caspase-dependent hepatocellular apoptosis discussed above is mediated by NF-*κ*B through a negative feedback loop. TNF-*α* induces NF-*κ*B activation, which negatively feedbacks to inhibit TNF-*α*-induced, caspase-dependent apoptosis in mammalian hepatocytes [21]. NF-*κ*B consists of homo- and heterodimers composed of the NF-*κ*B members (e.g., p50, p52, p65, and c-Rel), the most prominent being the p50–p65 heterodimer which strongly induces transcription of NF-*κ*B-responsive genes. NF-*κ*B dimers are held in an inactive state in the cytoplasm by their association with I*κ*B. After I*κ*B is phosphorylated and subsequently degraded by the proteasome, the liberated NF-*κ*B dimers enter the nucleus and initiate transcription of genes with *κ*B sites. Here, EMSA and Western blotting revealed that G-Rg1 treatment significantly lowered NF-*κ*B p65 DNA binding activity and expression in both G-Rg1 groups (Figure 3). This finding is consistent with the significantly reduced TNF-*α* and caspase-8/3 levels found in both G-Rg1 groups.

There are several limitations to this study. First, only changes in caspase-3 and caspase-8 expression were assayed as downstream markers of TNF-*α*-induced hepatocellular apoptosis; future studies should seek to analyze more downstream target proteins in order to better validate these findings. Second, NF-*κ*B p65 was the only transcription factor assayed by EMSA here; future studies should aim to assess the DNA binding activity of other transcription factors activated or inhibited by TNF-*α* signaling in mammalian hepatocytes. Third, although we used an Epstein-Barr nuclear antigen- (EBNA-) based control system in the EMSA, we failed to use recombinant NF-*κ*B protein as an additional control in the EMSA. Finally, only specific competition was used in the EMSA when both specific and nonspecific competition should have been used.

In conclusion, this study indicates that G-Rg1 significantly reduces liver damage in a murine ALF model through inhibiting TNF-*α*-induced, caspase-dependent hepatocellular apoptosis. These results support the further investigation of G-Rg1 as a therapeutic candidate for ALF.

## Figures and Tables

**Table 1:** Survival rate in 4 groups.

Group	*N*	Death	Survival	Survival rate
Control	40	0	40	100%
ALF	40	18	22^*^	55%
Treatment a	40	10	30	75%
Treatment b	40	8	32^*^	80%

ALF: acute liver failure model.

^*^Chi-square test, df = 3, chi2 = 5.6980, *P* = 0.017 < 0.05.

**Table 2:** The plasma levels of AST, ALT, TB, TNF-*α*, IL-6, and IL-1.

Group	AST (U/L)	ALT (U/L)	TB (mg/dL)	TNF-*α* (pg/mL)	IL-6 (pg/mL)	IL-1*β* (pg/mL)
ALF	647.16 ± 123.33^b^	205.16 ± 50.82^b^	1.57 ± 0.27^b^	4135.91 ± 257.30^b^	2152.64 ± 486.24^b^	1739.55 ± 368.4^b^
Ta	512.01 ± 216.50^a^	71.01 ± 16.49^a^	1.12 ± 0.09^a^	2573.05 ± 207.67^a^	1287.57 ± 327.20^a^	1138.9 ± 354.92^a^
Tb	398.54 ± 121.59^a^	52.54 ± 18.33^a^	1.12 ± 0.25^a^	2646.65 ± 215.43^a^	1232.23 ± 444.56^a^	1235.1 ± 272.53^a^
CON	183.67 ± 27.57^a^	17.67 ± 2.38^a^	0.54 ± 0.17^a^	2118.53 ± 129.48^a^	809.83 ± 119.91^a^	332.97 ± 61.52^a^

^a^ *P* < 0.05 compared with ALF group; ^b^ *P* < 0.05 compared with CON group.
